# Dacarbazine-Loaded Targeted Polymeric Nanoparticles for Enhancing Malignant Melanoma Therapy

**DOI:** 10.3389/fbioe.2022.847901

**Published:** 2022-02-17

**Authors:** Wei Xiong, Zhengdong Guo, Baoyan Zeng, Teng Wang, Xiaowei Zeng, Wei Cao, Daizheng Lian

**Affiliations:** ^1^ Department of Plastic and Burn Surgery, Huazhong University of Science and Technology Union Shenzhen Hospital, Shenzhen, China; ^2^ Graduate School at Shenzhen, Tsinghua University, Shenzhen, China; ^3^ Department of Radiation Oncology, Shenzhen People’s Hospital The Second Clinical Medical College, Jinan University, The First Affiliated Hospital, Southern University of Science and Technology, Shenzhen, China

**Keywords:** cancer nanocarriers, targeted drug delivery, controlled release, copolymer, malignant melanoma

## Abstract

Dacarbazine (DTIC) dominates chemotherapy for malignant melanoma (MM). However, the hydrophobicity, photosensitivity, instability, and toxicity to normal cells of DTIC limit its efficacy in treating MM. In the present study, we constructed star-shaped block polymers nanoparticles (NPs) based on Cholic acid -poly (lactide-*co*-glycolide)-*b*-polyethylene glycol (CA-PLGA-*b*-PEG) for DTIC encapsulation and MM targeted therapy. DTIC-loaded CA-PLGA-*b*-PEG NPs (DTIC-NPs) were employed to increase the drug loading and achieve control release of DTIC, followed by further modification with nucleic acid aptamer AS1411 (DTIC-NPs-Apt), which played an important role for active targeted therapy of MM. *In vitro*, DTIC-NPs-Apt showed good pH-responsive release and the strongest cytotoxicity to A875 cells compared with DTIC-NPs and free DTIC. *In vivo* results demonstrated that the versatile DTIC-NPs-Apt can actively target the site of MM and exhibited excellent anti-tumor effects with no obvious side effects. Overall, this research provided multi-functional NPs, which endow a new option for the treatment of MM.

## Introduction

Malignant melanoma (MM) is one of the most life-threatening malignancies (Li et al., 2015; Liu et al., 2017). Although melanoma rarely occurs, accounting for only 5% of skin cancers, the survival rate of patients is low due to its rapid recurrence, high multidrug resistance, and easy metastasis. Specifically, the average survival time of patients with MM is about 8–9 months, and overall survival rate is less than 15% with 3 years (Balch et al., 2009). Although many studies have explored treatment of MM (Tagne et al., 2008; Li and Han, 2020), few have obtained satisfactory results. Surgery, chemotherapy, and immunotherapy are the most common methods for treatment (Chapman et al., 2011; Hamid et al., 2013; Koyama et al., 2016). Among them, chemotherapy occupies an important position in the comprehensive treatment of tumor (Hafeez and Kazmi, 2017; Yuan et al., 2017). Dacarbazine (DTIC) is the only US Food and Drug Administration (FDA)-approved chemotherapeutic agent for melanoma, it is also the first-line drug for MM (Chapman et al., 1999; Tarhini and Agarwala, 2006; Bei et al., 2009). DTIC is an alkylating agent that kills tumor cells by destroying DNA (Eigentler et al., 2011; Demetri et al., 2017). However, DTIC has limited efficacy in treating melanoma due to its hydrophobicity and short half-life (Tagne et al., 2008; Koyama et al., 2016) In addition, DTIC has photosensitivity and instability, which increase the difficulty of drug storage and transportation. Moreover, DTIC has non-specific toxicity to normal cells, which is a common defect of chemotherapy drugs (Almousallam et al., 2015). Therefore, it is very necessary to develop an effective platform to reduce toxic side effects and improve the cytotoxicity to MM cells, thereby inhibiting further metastasis and recurrence of metastatic MM.

Targeted therapy of tumors opened up a new world for chemotherapy. It is considered to be the most promising method in cancer treatment. In recent years, new tumor-targeted strategies have emerged, such as tumor-targeted drug therapy (Jin et al., 2021; Kerns et al., 2021; Li et al., 2021; Li et al., 2021), immunotherapy (Chen et al., 2021; Lu et al., 2021; Pei et al., 2021), gene therapy (Chada et al., 2015; Wang et al., 2021; Wang et al., 2021), virus therapy (Kemler et al., 2021; Wan et al., 2021; Zhang et al., 2021), and cell-based targeted therapy (Collet et al., 2016). Among them, tumor active targeted drug therapy based on nanoparticles (NPs) drug delivery systems achieved the most success. The targeted NPs utilize specific interaction between receptors expression on the surface of target cells and ligands conjugated with the NPs, specifically to deliver drug to tumor cells and organs, significantly improve drug concentration in the target area and effectively reduce side effects to normal tissues (Ashley et al., 2011; Bao et al., 2012; Dahlberg et al., 2015; Li et al., 2021). Moreover, versatile NPs could improve the stability of drugs and control release of cargos (Berger et al., 2015; Gong et al., 2019). Nucleolin is a protein widely present in the nucleus, cytoplasm, and cell membrane of many kinds of cells (Kabirian-Dehkordi et al., 2019). Interestingly, nucleolin is more abundantly expressed on the surface of tumor cell membranes than normal cells, which means that nucleolin can be a potential therapeutic target for anti-tumor (Koutsioumpa and Papadimitriou, 2014; Palmieri et al., 2015; Romano et al., 2018). Increasing interest in nucleolin anti-tumor research has led to the development of several small molecule antagonists (Kabirian-Dehkordi et al., 2019). Aptamer has attracted the most interest in the field of tumor targeted treatment due to its high affinity, strong specificity, no significant immunogenicity, small molecular size, and easy modification. Nucleic acid aptamer AS1411, the first aptamer approved by the FDA, was used in various cancer clinical treatment trials. AS1411 can specifically bind to nucleolin, which was highly expressed on the surface of tumor cells, to achieve the purpose of targeted therapy (Destouches et al., 2011; Ding et al., 2011). However, AS1411 was observed to be effective on partial tumors in clinical trials and suffer from its rapid clearance from the blood (Kabirian-Dehkordi et al., 2019). Therefore, the AS1411 conjugated to the NPs surface would improve its stability in the blood and endow NPs the ability to target tumor cells, maximizing anti-tumor effect.

With the development of nano-targeting technology, a variety of targeted NPs have been reported for the delivery of DTIC (Bei et al., 2009; Ding et al., 2011; Ding et al., 2011; Kakumanu et al., 2011). Yet, there are extremely rare drug carriers with targeted functions *in vivo* that have been successfully used in clinical practice because of following obstacles. First, the capacity of ligands which bind to these drug carriers is not enough to effectively recognize the target. Second, the binding stability between ligands and drug carriers is not strong enough to achieve systemic circulation, and is unable to reach the target site. Third, the speed and strength of ligands to bind to the target are insufficient after delivery to the destination. Obviously, there is an urgent need to design a carrier with multiple active sites, which can combine multiple ligands and drugs at the same time, to realize the synergy of targeted multivalency and drugs. Star-shaped block polymers based on Poly (lactide-*co*-glycolide) (PLGA) is a simple example of highly specific targeting carrier material. They are branched polymer with all branches extending from a single core, which endow them a higher drug loading capacity and encapsulation efficiency than linear polymers with the same molar mass (Nguyen-Van et al., 2011; Ma et al., 2013; Tao et al., 2014; Peng et al., 2018; Liu et al., 2019). Thus, they can flexibly guide target molecules to a destination in the complex environment of the body. Polyethylene glycol (PEG), a water-soluble polymer, was often introduced to PLGA in order to improve hydrophilicity of PLGA, to further meet the needs of drug delivery carriers (Yildiz and Kacar, 2021). In the process of PLGA star-shaped block polymers synthesis, the most commonly used core is cholic acid (CA). CA is composed of a steroid unit with three hydroxyl groups and one carboxyl group, which can be selected as the poly-hydroxy initiator. Furthermore, CA is the main bile acid in the body. This biological source can synthesize copolymers based on CA due to its good biocompatibility (Janvier et al., 2013; Su et al., 2017).

In the present study, CA-PLGA-*b*-PEG nanocarriers were constructed to encapsulate DTIC, and then modified with aptamer for targeted therapy of MM. The star-shaped block copolymer CA-PLGA-*b*-PEG was prepared by the core-first method, and NPs were fabricated by an improved nanoprecipitation method. Morphology and drug loading of the NPs were characterized and the antitumor effect of the NPs was evaluated both *in vitro* and *in vivo*. The results show the NPs exhibited an excellent anti-tumor effect, providing a new option for MM patients.

## Results and Discussions

### Synthesis and Characterization of Star-Shaped Copolymer CA-PLGA-b-PEG-COOH

**FIGURE 1:**
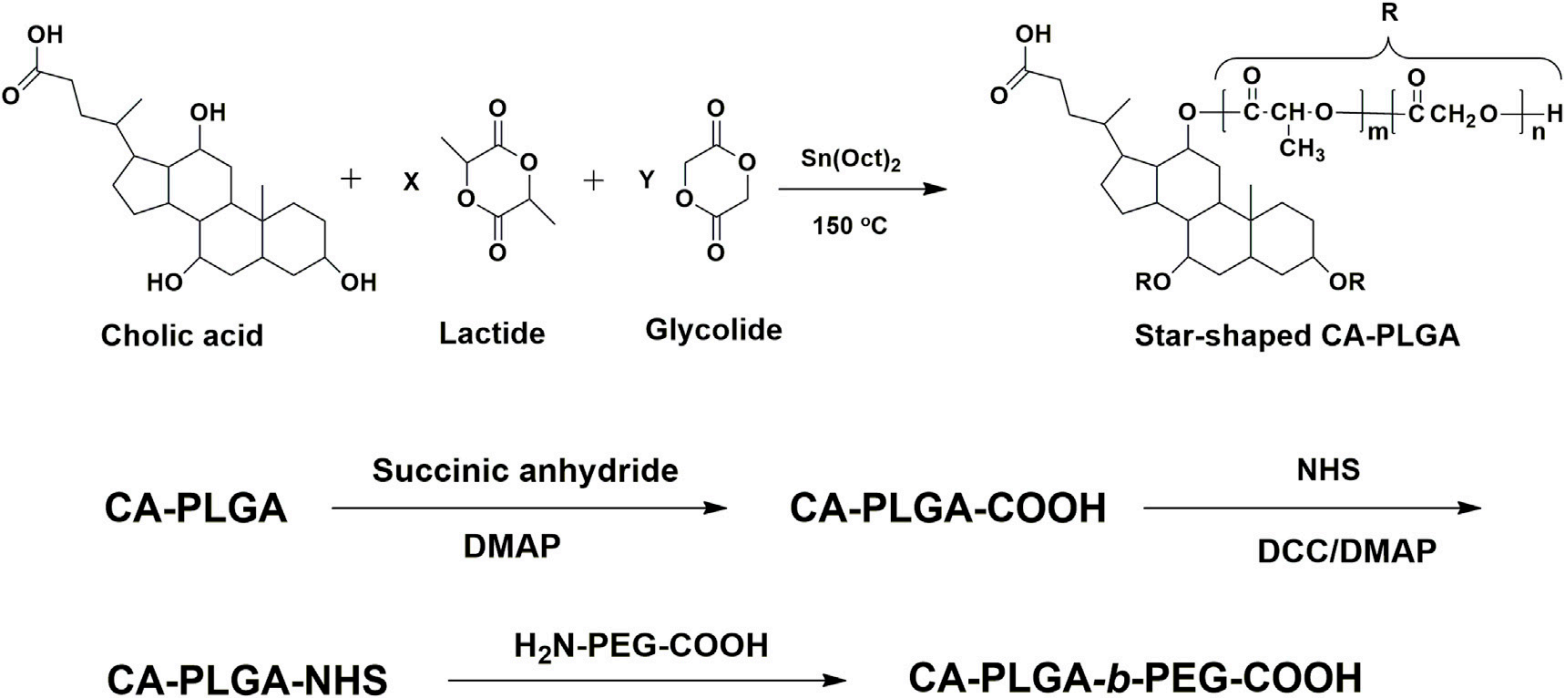
Schematic representation of synthesis of amphiphilic block copolymer CA-PLGA-*b*-PEG-COOH.

The chemical reaction scheme for synthesis of star-shaped polymer CA-PLGA-*b*-PEG-COOH was shown in Figure 1 The CA-PLGA-*b*-PEG-COOH with a well-defined three-branched structure were prepared successfully. The gel permeation chromatography (GPC) molecular weight *M*_n_ of CA-PLGA and CA-PLGA-*b*-PEG-COOH are 13,265 and 20,872, respectively. The results of GPC molecular weight could prove the CA-PLGA-*b*-PEG-COOH was synthesized successfully by the coupling reaction between CA-PLGA-COOH and NH_2_-PEG_2k_-COOH.

**FIGURE 2:**
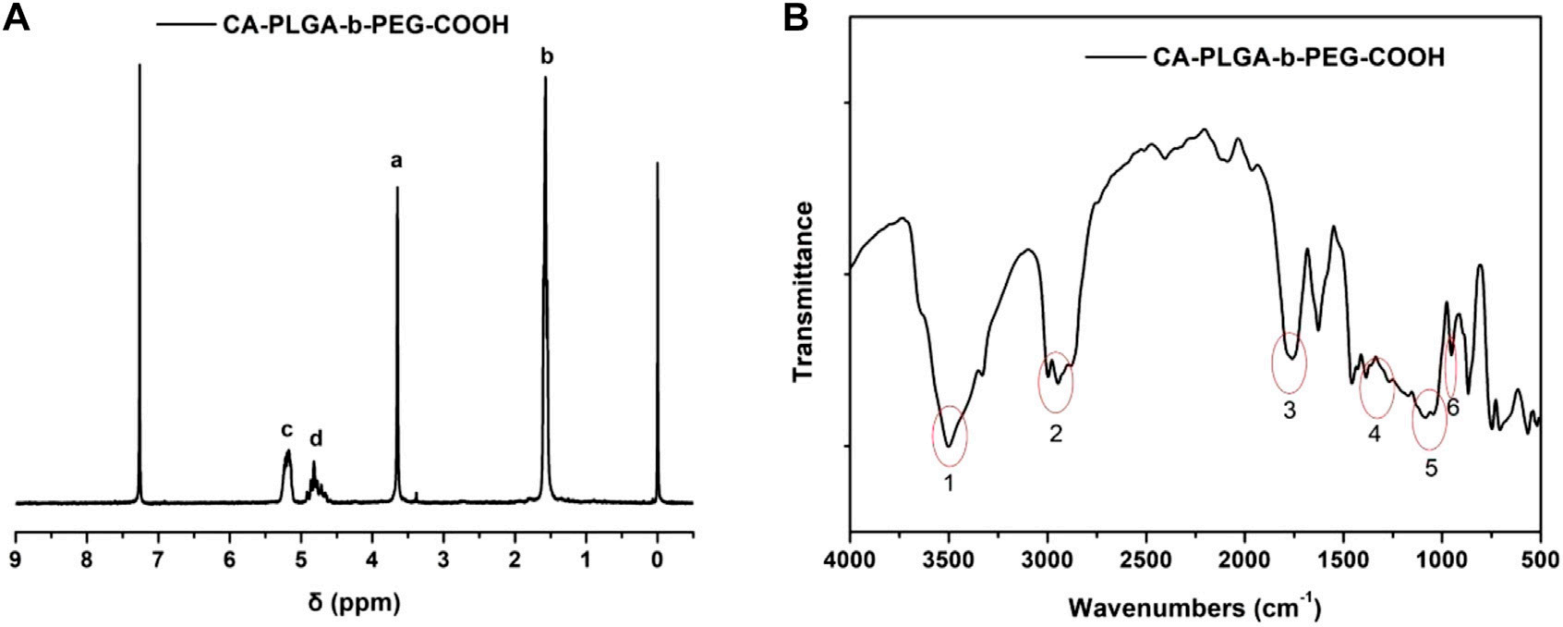
Typical characterization of copolymer CA-PLGA-*b*-PEG-COOH **(A)** ^1^H NMR spectra; **(B)** FT-IR spectra.

For star-shaped copolymer CA-PLGA-*b*-PEG-COOH, the typical ^1^H NMR signals from PEG and monomers lactide (LA) and glycolide (GA) repeating units can be observed. As shown in Figure 2A of ^1^H NMR (CDCl_3_), four characteristic signals can be observed: peak a (*δ* = 3.63 ppm, PEG repeating unit: -C**H**_2_C**H**_2_O-), b (*δ* = 1.60 ppm, LA repeating unit: -CHC**H**_3_), c (*δ* = 5.19 ppm, LA repeating unit: -C**H**CH_3_), and d (*δ* = 4.81 ppm, GA repeating unit: -C**H**_2_-). As shown in spectra, we can further demonstrate that the successful coupling of star-shaped CA-PLGA-COOH and NH_2_-PEG_2k_-COOH, and the star-shaped copolymer CA-PLGA-*b*-PEG-COOH was prepared successfully.

FT-IR spectroscopy was adopted for further confirming the structure and successfully synthesizing CA-PLGA-*b*-PEG-COOH. As shown in Figure 2B, the obvious characteristic peak at 1750 cm^−1^ (peak 3) could be ascribed to the presence of a carbonyl group in CA-PLGA. The stretching vibrations peak at 1,115–1,025 cm^−1^ (peak 5) could be ascribed to the presence of a C-O-C in PEG. The broad absorbance between 3,010 and 2,850 cm^−1^ (peak 2) corresponded to the stretching vibrations of C-H. The characteristic peak at 1, 2, 4, and 6 could be ascribed to the presence of a carboxy group (COOH) in the copolymer of CA-PLGA-*b*-PEG-COOH.

### Preparation and Characterization of Nanoparticles

**FIGURE 3:**
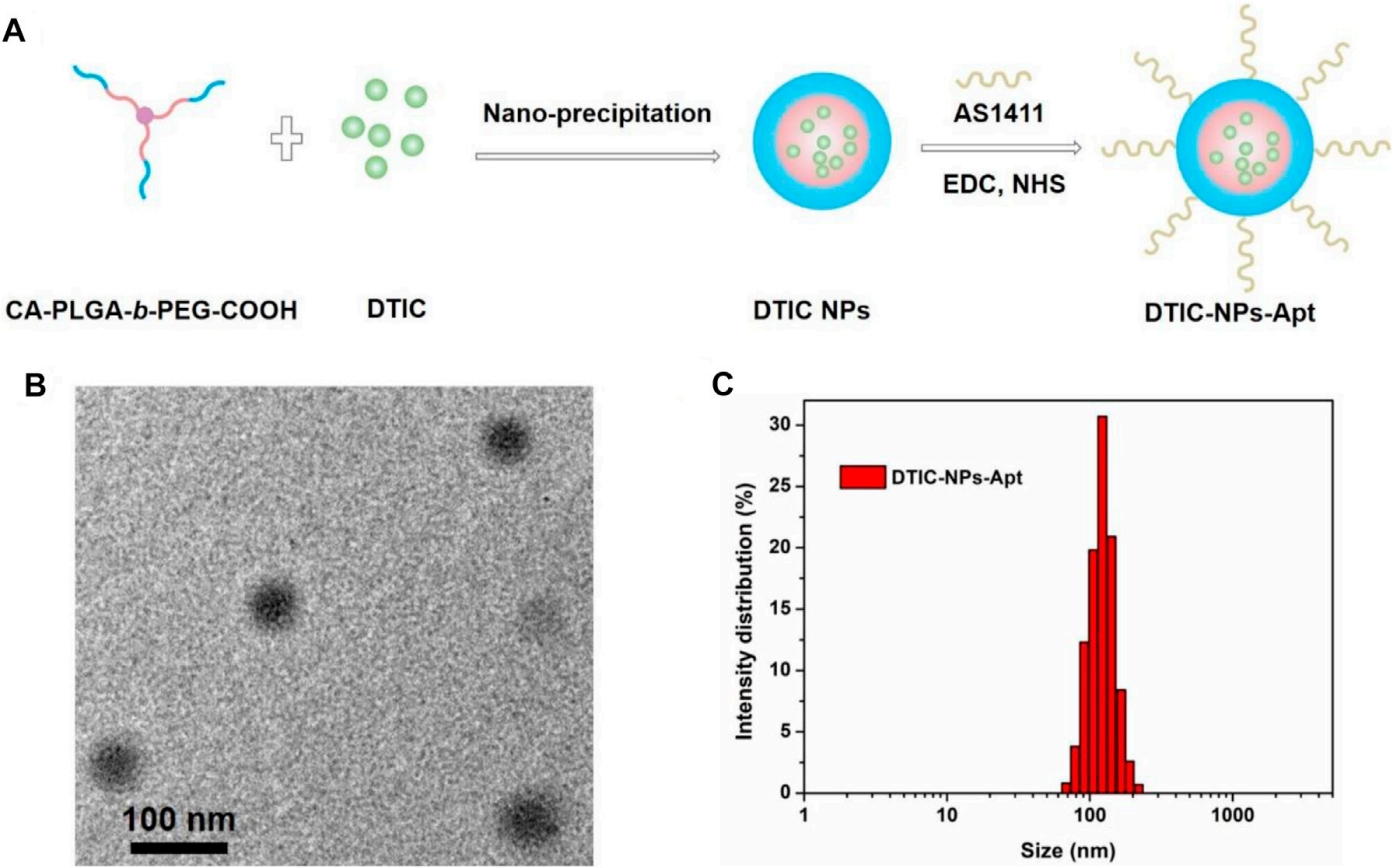
**(A)** Schematic illustration of the preparation technics for the targeted DTIC-NPs-Apt; **(B)** TEM image of DTIC-NPs-Apt; **(C)** DLS size distribution of DTIC-NPs-Apt.

DTIC-NPs was prepared by a modified nanoprecipitation method, and the synthesis process is shown in Figure 3A. Specifically, DTIC and copolymer are dissolved in a mixed solvent of acetone and methanol to form an organic solution. Then, the mixed solvent was added dropwise to the aqueous solution which contained emulsifier D-a-tocopheryl polyethylene glycol 1,000 succinate (TPGS). During this process, PLGA in the copolymer precipitates due to its hydrophobicity, resulting in the formation of drug loaded CA-PLGA-*b*-PEG NPs spontaneously. Finally, the organic solvent was evaporated by stirring overnight, and then, CA-PLGA-*b*-PEG NPs were centrifuged, washed, and lyophilized. For DTIC-NPs-Apt, EDC and NHS were utilized as catalysts to couple aptamer AS1411 to DTIC-NPs.

Stability, cumulative release of drug, cellular uptake, and *in vivo* biodistribution of NPs were mainly affected by its particle size and surface properties. The particle sizes and size distributions of the DTIC NPs and DTIC-NPs-Apt were studied by dynamic light scattering (DLS) method, and the results are shown in Table 1. The average diameter of DTIC NPs and DTIC-NPs-Apt was 116.3 ± 5.2 nm and 125.9 ± 4.1 nm, respectively. The average diameter of DTIC-NPs-Apt was slightly larger than DTIC NPs, which is because aptamer AS1411 was introduced into the surface of DTIC NPs. The nanoparticles used as drug delivery system possess high cellular uptake, more desirable biodistribution, and preferentially accumulate at the tumor site because of the enhanced permeability and retention (EPR) effect. Furthermore, nanoparticles can reduce tumor resistance to a certain extent. The hydrated particle size of both NPs is in a range that is suitable for cell uptake, which would boost the NPs to be passively targeted to the tumor tissue through the EPR effect, and more accumulated in the tumor tissue. Figure 3B exhibited the result of DTIC-NPs-Apt transmission electron microscopy (TEM), and the DTIC-NPs-Apt were spherical shaped. The average diameter of DTIC-NPs-Apt presented in TEM was about 70 nm, which was significantly reduced compared to the average diameter measured by DLS. This difference may be attributed to a shrinkage while the DTIC-NPs-Apt are in dry state during the TEM characterization. Figure 3C shows the size distribution of the DTIC-NPs-Apt. Polydispersity index of both NPs was less than 0.2, indicated the particle size is uniform, which was beneficial to deliver the cargos. The drug loading of DTIC NPs and DTIC-NPs-Apt was 8.72 and 7.64%, respectively. It was noted that the aptamer AS1411 functionalization dose not influence the drug loading content of NPs. These results suggested that the successful construction of DTIC-NPs-Apt with suitable average diameter, uniform particle size, and high drug loading.

**TABLE 1:** Characterization of DTIC-NPs and DTIC-NPs-Apt (Mean ± SD, *n* = 3).

Samples	Size (nm)	PDI	ZP (mV)	LC (%)	EE (%)
DTIC-NPs	116.3 ± 5.2	0.128	−26.4 ± 3.9	8.72	88.53
DTIC-NPs-Apt	125.9 ± 4.1	0.115	−15.7 ± 2.6	7.64	N/A

PDI, polydispersity index; ZP, zeta potential; LC, loading content; EE, encapsulation efficiency; N/A, not applicable.

### Stability of DTIC-NPs and DTIC-NPs-Apt

**FIGURE 4:**
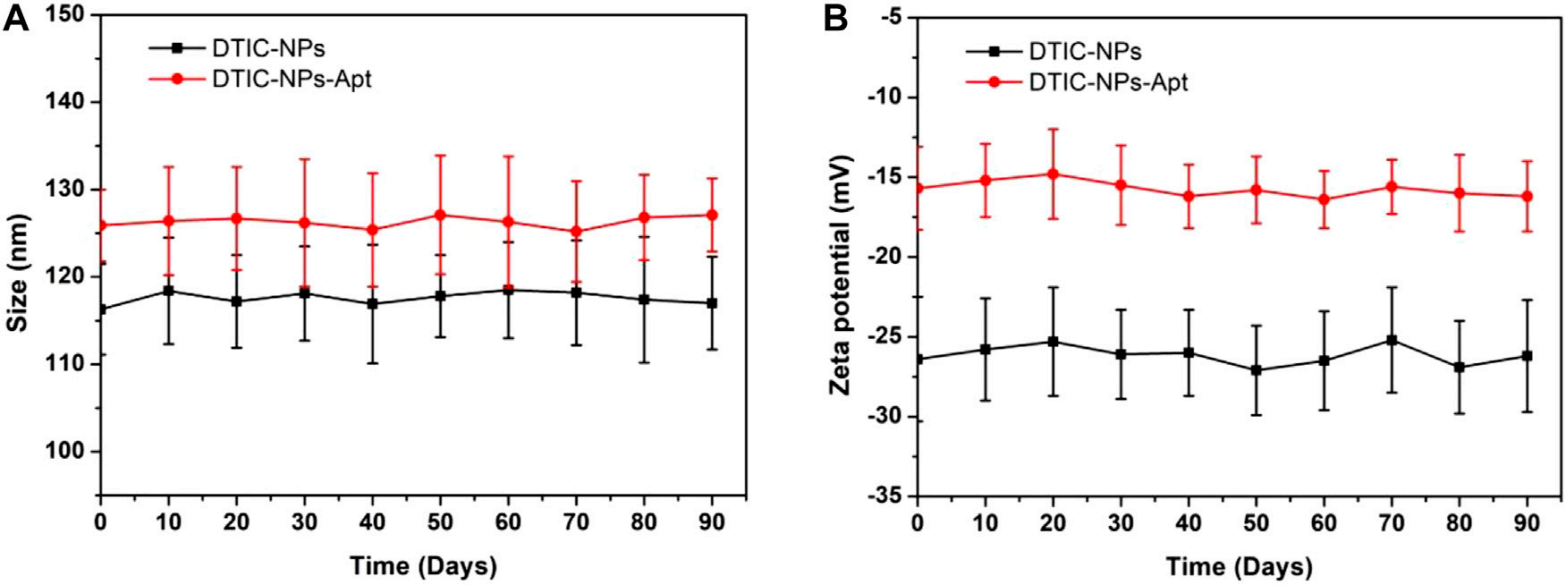
*In vitro* stability of DTIC-NPs and DTIC-NPs-Apt. **(A)** Particle size and **(B)** zeta potential during 90 days of storage, respectively.

Zeta potential can detect the mutual repulsion between NPs, which plays an important role in maintaining the physical stability of NPs. The zeta potential of DTIC NPs and DTIC-NPs-Apt are presented in Table 1, which were -26.4 ± 3.9 mV and -15.7 ± 2.6 mV, respectively. Both NPs behaved negatively charged, which is due to the ionized carboxyl groups of polylactic acid and polyglycolic acid segments. The absolute value of modified DTIC-NPs-Apt zeta potential was reduced, which indicated that modified aptamer AS1411 on the NPs surface has a surface charge shielding effect. To further assess the stability of two kinds of NPs, we monitored the changes in the size and zeta potential of the DTIC NPs and DTIC-NPs-Apt during storage. The results are shown in Figure 4; no obvious change was observed in particle size and zeta potential during 3 months. These data show NPs exhibit excellent stability, which was important to achieve clinical translation.

### *In Vitro* Drug Release Profiles

**FIGURE 5:**
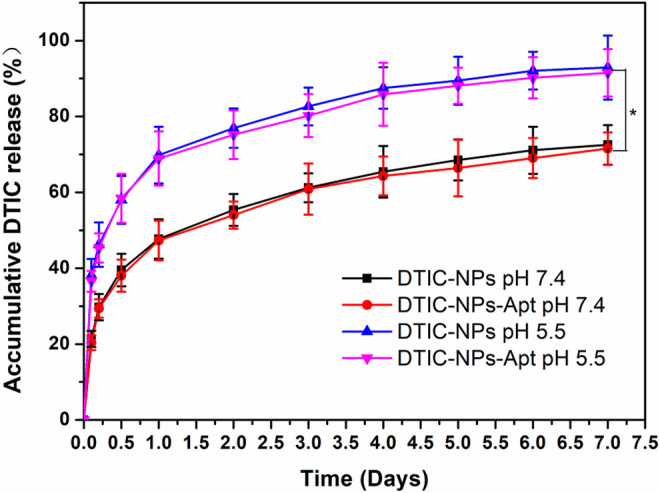
*In vitro* drug release profiles of the DTIC-NPs and DTIC-NPs-Apt at pH 5.5 and 7.4, respectively. ∗ *p* < 0.05.

Figure 5 displayed the cumulative drug release profiles of the DTIC-NPs and DTIC-NPs-Apt in PBS (pH 7.4) for 7 days. During the first day, the initial burst release of DTIC-NPs and DTIC-NPs-Apt was found to be 47.7 and 47.3%, respectively. However, both NPs exhibited continuous, steady release patterns in the following few days. After 1 week, the accumulative drug releases of DTIC-NPs and DTIC-NPs-Apt reached 72.5 and 71.6%, respectively. These data indicated that both the NPs exhibited a typically biphasic pattern release with an initial burst. Because DTIC was absorbed on the surface or entrapped weakly by NPs, DTIC was quickly released in the initial day. Subsequently, DTIC was released with a sustained behavior dominantly attributed to the diffusion of the cargo from the rigid core or hydrophobic inner shell. *In vitro* cumulative drug release profiles of DTIC-NPs and DTIC-NPs-Apt at pH 5.5 PBS were also carried out. The release behavior of both NPs at pH 5.5 was similar to that at pH 7.4, but the release rate is faster. The initial burst release of DTIC-NPs and DTIC-NPs-Apt at pH 5.5 was found to be 69.8 and 69.0%, respectively. The cumulative release of DTIC after 7 days was 92.9 and 91.5%, respectively. The reason may be because the structure of the NPs is destroyed in the acidic environment of pH 5.5. Based on the characteristics of acid response, the DTIC loaded in NPs can be better released in the acidic environment of tumors, promoting anti-tumor effect.

### *In vitro* Cell Viability of NPs

**FIGURE 6:**
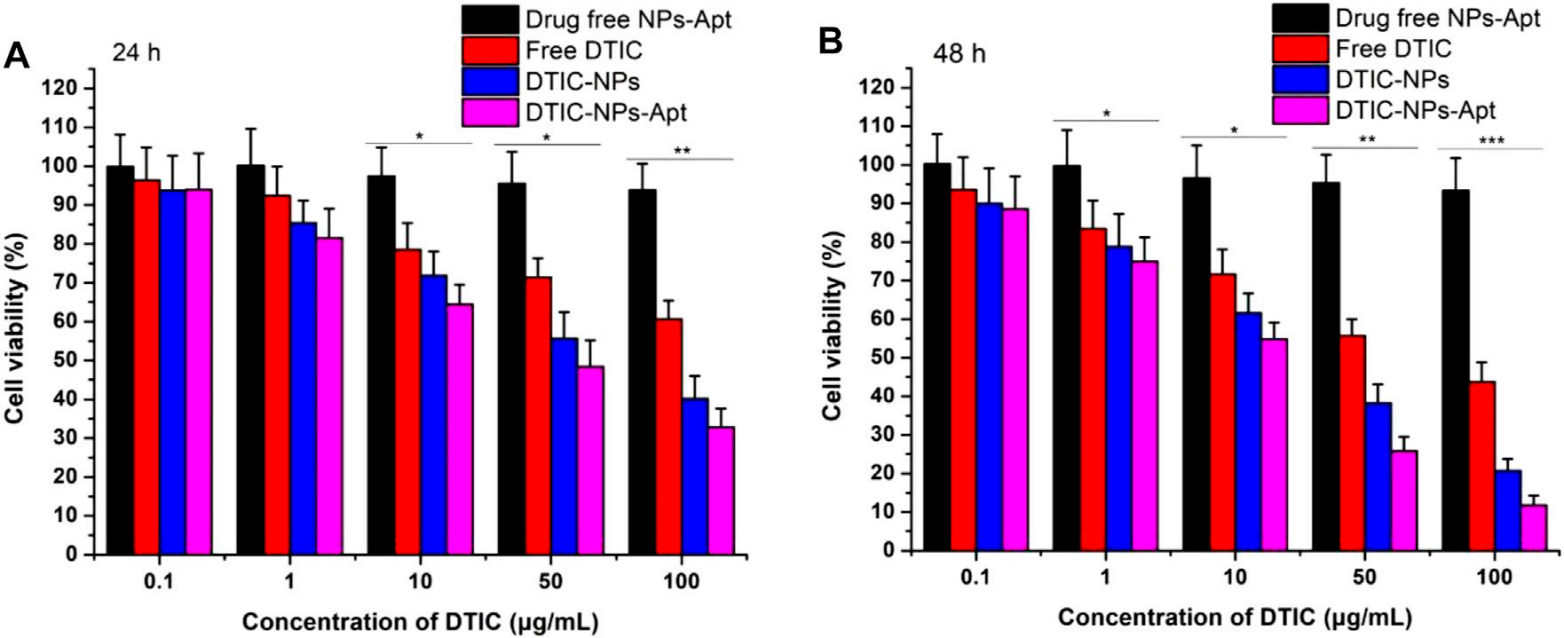
Viability of human melanoma A875 cells cultured with drug free NPs-Apt, free DTIC, DTIC-NPs, and DTIC-NPs-Apt at equivalent drug concentrations ranging from 0.1 to 100 μg/ml. The amount of drug free NPs-Apt was the same as that of the NPs, *n* = 3. **(A)** 24 h; **(B)** 48 h ∗ *p* < 0.05; ∗∗ *p* < 0.01; ∗∗∗ *p* < 0.001 indicate significant difference compared with drug free NPs-Apt.

Human melanoma A875 cells were cultured to evaluate cytotoxicity of the NPs *in vitro*. Free DTIC, DTIC-NPs, and DTIC-NPs-Apt with same drug concentrations ranging from 0.1 to 100 μg/ml were tested. Drug free NPs-Apt with equivalent NPs dosage were used as well. As shown in Figure 6, blank NPs-Apt at different concentrations seem not to have exhibited significant cytotoxicity at a different time, which suggested NPs possess nontoxic and excellent biocompatibility. The cytotoxicity of free DTIC and DITC-loaded NPs showed dose-dependent and time-dependent behavior. In addition, DTIC-NPs-Apt exhibited the strongest cytotoxicity. After incubation with NPs for 24 h with DTIC concentration of 100 μg/ml, viability of A875 cells was 60.6% for free DTIC, 40.1% for DTIC-NPs, and 32.8% for DTIC-NPs-Apt. However, after incubating A875 cells with NPs for 48 h, the cytotoxicity of DTIC-NPs-Apt was 11.8%, significantly lower than DTIC-NPs, which was 20.7%. This result was attributed to the modification of aptamer AS1411 on the DTIC-NPs-Apt, which could target to nucleolin. Nucleolin was highly expressed on the surface of A875 cells, DTIC-NPs-Apt with active targeting function could enhance cellular uptake of NPs, therefore improving cytotoxicity to A875 cells.

The IC_50_ values of free DTIC, DTIC-NPs, and DTIC-NPs-Apt against A875 cells were also quantified. IC_50_ values of A875 cells treated with three DTIC formulations for 24 and 48 h are summarized in Supplementary Table S1. Compared with free DTIC, DTIC NPs can significantly reduce the value of IC_50_, which benefits from the continuous release effect of nanocarrier on DTIC. Especially the IC_50_ of DTIC-NPs-Apt group was less than half of DTIC-NPs group, demonstrating that after active targeting group-aptamer AS1411 modified, DTIC-NPs-Apt showing strongest cytotoxicity of A875 cells.

### *In Vivo* Antitumor Efficacy

**FIGURE 7:**
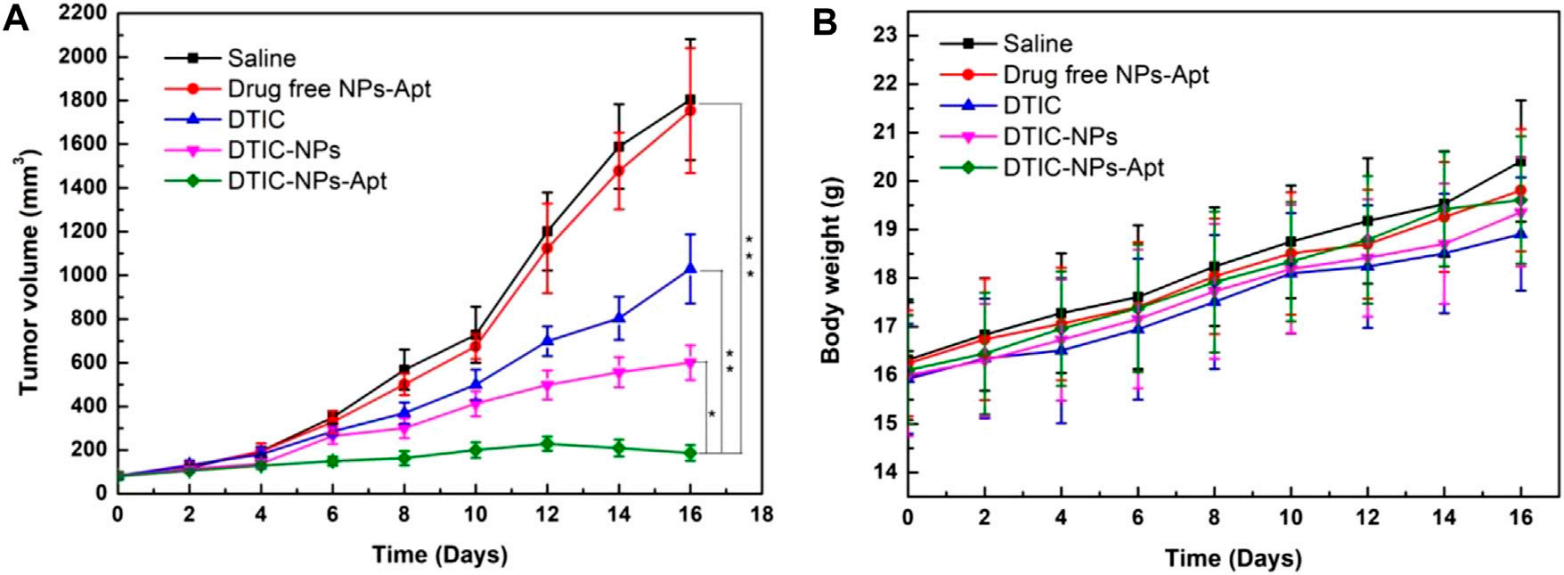
Antitumor efficacy of targeted DTIC-NPs-Apt in comparison with saline, drug free NPs-Apt, free DTIC, and DTIC-NPs (*n* = 5). **(A)** Tumor growth curve of the SCID nude mice bearing human melanoma A875 cells xenograft. **(B)** Animal body weight of the nude mice in different groups after treatment at different time intervals. ∗ *p* < 0.05; ∗∗ *p* < 0.01. ∗∗∗ *p* < 0.001 indicate significant difference compared with DTIC-NPs-Apt.

Due to the excellent cytotoxicity to A875 cells *in vitro*, DTIC-NPs-Apt bring a potential hope to the treatment of MM. In this study, the antitumor effect of DTIC-NPs-Apt was also evaluated *in vivo*. Twenty-five tumor-bearing nude mice were employed and divided into five groups randomly. Then, five groups were injected with saline (control), blank NPs-Apt, free DTIC, DTIC-NPs, and DTIC-NPs-Apt through the tail vein, respectively. Every other day, we recorded the tumor volume and weight of the mouse until the end of the treatment. Tumor growth curve of tumor-bearing mice was shown in Figure 7A. Compared with control group, three DTIC formulation groups significantly reduced tumor growth. Furthermore, DTIC-NPs-Apt group displayed the strongest ability to inhibit tumor growth over the free DTIC and DTIC-NPs groups. In general, when various formulations were injected into the tail vein, the surface modification of aptamer AS1411 (DTIC-NPs-Apt group) can actively target to MM cells to achieve high concentration aggregation of tumor sites, and release DTIC in response to the acidic environment of the tumor, resulting in the strongest anti-tumor effect. In the development of novel carriers, the side effects of carriers have received extensive attention. The body weight of the mice during the treatment was also recorded to evaluate the systemic toxicity of star-shaped nanocarriers constructed in this study. Body weights of all nude mice was presented in Figure 7B. All groups gained weight, which indicated the nanocarriers show no obviously systemic side effects on mice.

**FIGURE 8:**
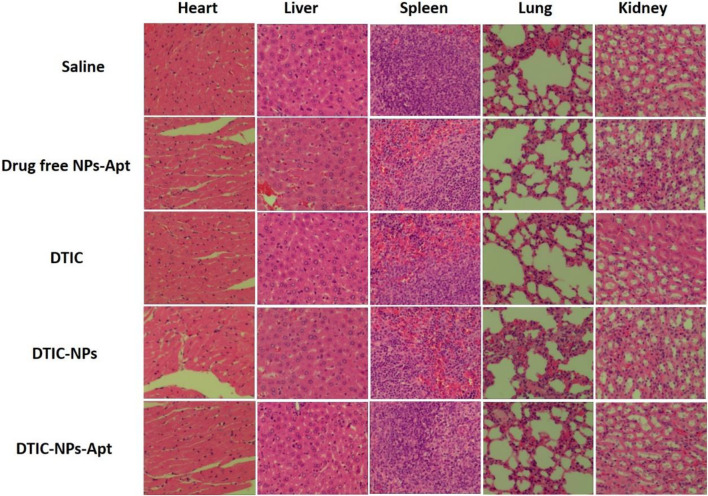
Representative tissue sections of mice stained with hematoxylin and eosin (H&E) after 16 days of treatment. For all the groups, no apparent tissue injury was observed in the tissues of heart, liver, spleen, lung, and kidney.

In order to further explore the toxic and side effects of the constructed nanocarriers, we used hematoxylin and eosin (H&E) staining to observe the changes in the heart, liver, spleen, lung, and kidney of mice after treatment. The results are shown in Figure 8. For all the groups, no apparent tissue injury was observed in the tissues of heart, liver, spleen, lung, and kidney. Therefore, all the results indicated that DTIC-NPs-Apt own excellent effects to treat MM with almost no toxic side effects. DTIC-NPs-Apt have the potential to provide a new treatment method for MM patients.

## Conclusion

In short, we have successfully constructed DTIC-NPs-Apt based on star-shaped block polymers and modified nucleic acid aptamer to realize active targeted therapy of MM. The appearance of DTIC-NPs-Apt was nearly spherical, and they had a suitable particle size range with a narrow size distribution and exhibited good stability. Moreover, DTIC-NPs-Apt displayed high drug loading of DTIC, showing controlled release and pH-response release behavior. Compared with DTIC-NPs or free DTIC, the modification of AS1411 and the controlled release effect of DTIC make the DTIC-NPs-Apt possess the best anti-tumor effect *in vivo* and *in vitro* without showing toxic side effects. In conclusion, DTIC-NPs-Apt have a potential for MM targeting therapy.

## Data Availability

The original contributions presented in the study are included in the article/Supplementary Material, further inquiries can be directed to the corresponding author.
