# Supplementary material for: Dacarbazine-Loaded Targeted Polymeric Nanoparticles for Enhancing Malignant Melanoma Therapy
Source: Front Bioeng Biotechnol. 2022 Feb 17;10:847901. doi: 10.3389/fbioe.2022.847901 (PMC8892180; doi:10.3389/fbioe.2022.847901)
Supplement: Supplementary file 1 [file DataSheet1.docx]

***Supporting Information***

**Dacarbazine-Loaded Targeted Polymeric Nanoparticles for Enhancing Malignant Melanoma Therapy**

Wei Xiong^1,*^, Zhengdong Guo^1^, Baoyan Zeng^1^, Teng Wang^2^, Xiaowei Zeng^2^, Wei Cao^2^ and Daizheng Lian^3^

*^1^* *Department of Plastic and Burn Surgery, Huazhong University of Science and Technology Union Shenzhen Hospital, Shenzhen 518052, China*

*^2^ Graduate School at Shenzhen, Tsinghua University, Shenzhen 518055, China;*

*^3^ Department of Radiation Oncology, Shenzhen People’s Hospital (The Second Clinical Medical College，Jinan University; The First Affiliated Hospital, Southern University of Science and Technology), Shenzhen 518020, China*

^*^ Correspondence:

Wei Xiong, wxiong68@email.szu.edu.cn

**Materials and methods**

*Materials*

D-α-tocopheryl polyethylene glycol 1000 succinate (TPGS), 3-(4,5-dimethylthiazol-2-yl)-2,5-diphenyltetrazolium bromide (MTT), cholic acid (CA), D,L-lactide (LA), glycolide (GA), stannous octoate (Sn(Oct)_2_), 1,3-diisopropylcarbodiimide (DCC), 4-(dimethylamino)pyridine (DMAP), 1-(3-dimethylaminopropyl)-3-ethylcarbodiimide hydrochloride (EDC·HCl), N-Hydroxysuccinimide (NHS), succinic anhydride (SA) and trifluoroacetic acid were purchased from Sigma-Aldrich (St. Louis, MO, USA). NH_2_-PEG_2k_-COOH was obtained by Shanghai Yare Biotech, Inc. (Shanghai, China). Dacarbazine (DTIC) was bought from Shanghai Yuanye Bio-Technology Co., Ltd (Shanghai, China). Aptamer AS1411 (NH_2_-5’-(GGTGGTGGTGGTTGTGGTGGTGG TGG)-3’, Apt-NH_2_) was provided by Sangon Biotech. (Shanghai, China). Acetonitrile and Dichloromethane were purchased from EM Science (HPLC grade, Mallinckrodt Baker, USA). All other chemicals of the highest quality were commercially available and used as received. Human melanoma cell line A875 was purchased from American Type Culture Collection (ATCC, Rockville, MD).

*Synthesis and characterization of star-shaped copolymer CA-PLGA-b-PEG-COOH*

Synthesis of the star-shaped block copolymer CA-PLGA-*b*-PEG-COOH was performed as described in the literature (Zeng et al., 2013).The chemical reaction scheme for synthesis of CA-PLGA-*b*-PEG-COOH was shown in Fig. 1. ^1^H NMR (Bruker AVANCE NEO 500M) was used to confirm the structure of synthesized CA-PLGA-*b*-PEG-COOH with CDCl_3_ used as a solvent. Molecular weight and molecular weight distribution were determined by gel permeation chromatography (Waters GPC analysis system with RI-G1362A refractive index detector, Waters Corp., Milford, MA, USA). FT-IR spectra were analyzed by Thermo Scientific Nicolet iS 50 spectrometer.

*Formulation of DTIC-loaded nanoparticles (DTIC-NPs) and aptamer targeted DTIC-loaded nanoparticles (DTIC-NPs-Apt)*

Dacarbazine-loaded CA-PLGA-*b*-PEG-COOH nanoparticles (DTIC-NPs) were prepared by a modified nanoprecipitation method according to the previous described (Ding et al., 2011; Gong et al., 2019). In brief, a pre-weighed amount of DTIC and 200 mg of copolymer CA-PLGA-*b*-PEG-COOH were dissolved in 20 mL mixture of acetone and methanol (4:1, v/v) acetone by vortexing and sonication. This mixture was dropwise added into 200 mL aqueous solution (including 0.03% TPGS) under stirring. The resulting suspension was then stirred uncovered overnight to remove mixed solvent acetone and methanol completely. The NPs suspension was centrifuged at 20,000 rpm for 20 min and then washed three times to remove the emulsifier TPGS and unencapsulated drug. Finally, the dispersed solution was lyophilized 2 days for further use. Drug free CA-PLGA-*b*-PEG-COOH NPs were prepared in a similar manner. The lyophilized NPs were redispersed in PBS before use.

Aptamer targeted DTIC-NPs-Apt were fabricated by coupling reaction of DTIC-NPs and aptamer in the presence of EDC and a catalytic amount of NHS. Drug free NPs-Apt were prepared in a similar manner.

*Size, zeta potential and morphology of the NPs*

The particle size and zeta potential were measured by Malvern Mastersizer 2000 (Zetasizer Nano ZS90, Malvern Instruments Ltd., UK). Before measurement, the freshly prepared nanoparticles were appropriately diluted. All measurements were measured at room temperature after equilibration for 10 min. The data were achieved with the average of three measurements.

The morphology of the NPs was observed by transmission electron microscopy (TEM, Tecnai G2 20, FEI Company, Hillsboro, Oregon, USA). NPs were dropped onto a copper grid coated carbon membrane, the grid was allowed to dry before characterization.

*Drug loading and drug encapsulation efficiency*

High-pressure liquid chromatography (HPLC) (LC 1200, Agilent Technologies, CA, USA) was used to measure the amount of DTIC as drug loading or encapsulation efficiency. Chromatographic separations were performed on a reversed phase-C18 column (4.6 × 200 mm, 5 μm, Agilent Technologies, CA, USA). Methanol/water (30/70, v/v) was used as eluent at a flow rate of 1ml/min. The column effluent was detected using a UV detector at λ_max_ of 319 nm. Drug loading content (LC) and entrapment efficiency (EE) were determined as follows. Briefly, 10 mg of drug loaded DTIC-NPs and DTIC-NPs-Apt were introduced into EP tube and were dissolved in 1 ml acetonitrile and diluted by 0.1 M citric acid. Meanwhile, the amount of DTIC in the solution was determined by HPLC. The measurement was performed in triplicate. The LC and EE of the DTIC-NPs and DTIC-NPs-Apt were calculated by the following equations, respectively.

$$\text{LC (\%) = }\frac{\text{Weight of DTIC in the NPs}}{\text{Weight of the NPs}}\text{ }\times\text{ 100\% }$$

$$\text{EE (\%) = }\frac{\text{Weight of DTIC in the NPs}}{\text{Weight of the feeding DTIC }}\text{ }\times\text{ 100\% }$$

*In vitro drug release profiles*

*In vitro* DTIC released from DTIC-NPs and DTIC-NPs-Apt was determined by HPLC using the same procedure as described above. In short, 5 mg freeze-dried DTIC-loaded NPs were dispersed in 1 ml of phosphate buffer solution at different pH values (pH 7.4 and 5.5, PBS, containing 0.1% w/v Tween 80). Tween 80 was used for increasing the solubility of DTIC in the PBS and avoiding the DTIC bind to the tube wall. The mixture then was put into a dialysis bag (MW cut off: 4000, Shanghai Sangon Biotechnology Co. China) and the bag was immersed in 20 ml of respective PBS in a tube. After that, tube was shaken in an orbital water bath at 200 rpm at 37 ℃. At designated time intervals, the release medium outside the dialysis bag was used for HPLC analysis and the dialysis bag was put into a new fresh PBS every day for 7 days. The cumulative release of DTIC was plotted against time.

*In vitro cytotoxicity*

Human melanoma cells A875 were seeded in 96-well plates at the density of 5×10^3^ viable cells per well in 100 μl of medium and incubated overnight. The cells were incubated with the free DTIC, DTIC-NPs, and DTIC-NPs-Apt at equivalent drug concentrations ranging from 0.1 to 100 μg/mL or the drug free NPs-Apt of the same NPs concentration for 24 and 48 h. At designated time intervals, the formulations were replaced with the DMEM containing MTT (5 mg/mL) and cells were then incubated for additional 4 h. MTT was aspirated off and DMSO was added to dissolve the formazan crystals. Absorbance was measured at 570 nm using a microplate reader. Untreated cells were taken as a negative control with 100% viability and cells without addition of MTT were used as blank to calibrate the spectrophotometer to zero absorbance. The inhibitory concentration IC_50_, the drug concentration at which inhibition of 50% cell growth was observed, was calculated by curve fitting of the cell viability vs drug concentration data.

*In vivo antitumor efficacy*

Female severe combined immunodeficient (SCID) mice (BALB/c-nu, 5~6 weeks old) were bought from Guangdong Medical Laboratory Animal Center (China). All the protocols for the proposed *in vivo* experiments were approved by the Administrative Committee on Animal Research in Shenzhen Nanshan People's Hospital (Huazhong University of Science and Technology Union Shenzhen Hospital). Guidelines of the institutional animal ethics committee were followed for *in vivo* experi­ments. Each mouse was subcutaneously injected with 100 μL of medium containing about 2×10^6^ A875 cells into the right axilla. Tumors were measured by a vernier caliper and its volume (V) was calculated as V=d^2^×D/2, where d and D are shortest and the longest diameter of the tumor in mm respectively. Treatment was performed when the volume reached approximately 80 mm^3^ (referred to as the 0th day). The tumor-bearing mice were divided into five groups randomly (*n* = 5) and subjected to tail intravenous injection with saline, drug-free NPs-Apt, free DTIC, DTIC-NPs, and DTIC-NPs-Apt with DTIC dosage of 10 mg kg^-1^. The injection was conducted every 4 days. The tumor volume and body weight of the treated-mice were recorded every other day until the end of the treatment. To further investigate the DTIC nanoplatform toxicity on the tissues, a histology study was also carried out. The tissues including heart, liver, spleen, lung and kidney from each group were isolated and fixed in 10% neutral buffered formalin. Finally, the tissues were embedded in paraffin followed by sectioning into about 4 μm slices, stained with hematoxylin and eosin (H&E) and observed by optical microscope.

*Statistical methodology*

All experiments were repeated at least three times unless otherwise stated. The values are presented as the mean ± standard deviation (SD). Statistical analysis was carried out by using SPSS 22.0 software for a one-way analysis of variance and subsequent Bonferroni test, with probability (*P*) less than 0.05 was considered statistically significant.

**Table S1**

IC_50_ values of free DTIC, DTIC formulation in DTIC-NPs and DTIC-NPs-Apt on A875 human melanoma cells following 24 and 48 and hours incubation, respectively (Mean ± SD, *n*=3).

| Incubation time (h) | IC_50_ (μg/mL) | | |
| --- | --- | --- | --- |
|  | Free DTIC | DTIC-NPs | DTIC-NPs-Apt |
| 24 | 341.1 ± 25.3 | 63.4 ± 5.1 | 30.5 ± 4.2 |
| 48 | 72.3 ± 6.6 | 13.4 ± 1.8 | 5.9 ± 0.6 |

REFERENCES

Ding, B.-y., Zhang, W., Wu, X., Wang, X., Fan, W., Gao, S., et al. (2011). Biodegradable methoxy poly (ethylene glycol)-poly (lactide) nanoparticles for controlled delivery of dacarbazine: Preparation, characterization and anticancer activity evaluation. *African Journal of Pharmacy and Pharmacology* 5, 1369–1377. doi: 10.5897/AJPP11.236

Gong, X., Zheng, Y., He, G., Chen, K., Zeng, X., and Chen, Z. (2019). Multifunctional nanoplatform based on star-shaped copolymer for liver cancer targeting therapy. *Drug delivery* 26, 595–603. doi: 10.1080/10717544.2019.1625467

Zeng, X., Tao, W., Mei, L., Huang, L., Tan, C., and Feng, S.-S. (2013). Cholic acid-functionalized nanoparticles of star-shaped PLGA-vitamin E TPGS copolymer for docetaxel delivery to cervical cancer. *Biomaterials* 34, 6058–6067. doi: 10.1016/j.biomaterials.2013.04.052
